# Mix and match strategies in revision total hip arthroplasty: a review of techniques and outcomes

**DOI:** 10.1007/s00402-026-06510-1

**Published:** 2026-09-25

**Authors:** Itay Ashkenazi, Farouk Khury, Sujith Konan

**Affiliations:** 1 The Ruth and Bruce Rappaport Faculty of Medicine, Technion - Israel Institute of Technology, Haifa, Israel; 2 University College London Hospitals NHS Foundation Trust, London, United Kingdom; 3 Rambam Health Care Campus, Haifa, Israel; 4 Tel-Aviv Sourasky Medical Center, Tel-Aviv, Israel

**Keywords:** Revision total hip arthroplasty; Aseptic revision hip arthroplasty; Mix and match revision techniques of hip arthroplasty; Surgical techniques revision arthroplasty

## Abstract

**Purpose:**

This review evaluates the clinical indications, surgical rationale, and outcomes of six targeted component-retention strategies in revision total hip arthroplasty (THA) designed to reduce operative morbidity and preserve host bone stock.

**Methods:**

This is a narrative review. A structured but non-systematic search of PubMed/MEDLINE and Scopus was used to identify contemporary clinical cohorts, joint registry data, and biomechanical analyses, with the evidence organised sequentially across acetabular and femoral subsites.

**Results:**

Acetabular retention of stable metal-on-metal shells with dual-mobility bearings minimizes surgical trauma and yields exceptionally low dislocation rates (0% to 4%). Cementing highly cross-linked polyethylene (XLPE) or dual-mobility liners into secure or revision cups successfully decouples biological fixation from alignment, ensuring high mid-term survivorship. On the femoral side, trunnion adapter sleeves protect the head-neck junction during isolated bearing exchanges, demonstrating reliable 10-year survivorship (85%). For unavoidable stem extractions with sound cement mantles, cement-in-cement revision offers a rapid pathway; utilizing high-fatigue-resistant cobalt-chromium alloys instead of stainless steel structurally mitigates the rare risk of cantilever stem fracture. Lastly, for uncontained bone loss or profound osteopenia, extended long cemented stems and allograft-stem composites bypass major defects to provide immediate biological and mechanical rigidity.

**Conclusion:**

In appropriately selected patients, targeted component retention appears to offer a bone-preserving alternative to wholesale hardware explantation, although the supporting evidence is predominantly observational.

## Introduction

The primary objective of revision total hip arthroplasty (THA) is to definitively address the mode of implant failure while minimizing operative morbidity and preventing subsequent re-revisions. Fulfilling these objectives is highly challenging, as revision THA carries an elevated risk of perioperative complications and functionally inferior patient-reported outcome measures (PROMs) when compared to primary procedures [1]. Despite this historical discrepancy in outcomes, patient expectations regarding long-term pain relief, functional recovery, and implant longevity remain disproportionately high [2].

To bridge this gap by simplifying the surgical workflow and reducing morbidity without compromising final PROMs, modern revision strategies frequently employ the targeted retention of well-fixed components. This pragmatic approach combines retention of stable host implants with modern modular or cemented adaptations.

This review evaluates six established techniques for component retention while revising the aseptic failed hip replacement component (i) retaining metal-on-metal (MoM) shells with dual-mobility bearings, (ii) cementing new bearings into existing revision shells, (iii) utilizing trunnion adapters on retained stems, (iv) performing cement-in-cement femoral revisions, (v) deploying long cemented stems, and (vi) constructing allograft-stem composites. The aim of this study is to describe the indications, surgical rationale, and clinical outcomes of these techniques to guide operative decision-making. These six techniques were selected because each shares a common principle, namely the deliberate retention of a well-fixed component or an intact cement mantle in preference to its removal; each is in established clinical use and supported by published outcome series rather than isolated case reports; and collectively they span both the acetabular and femoral sides across the spectrum of revision complexity, from isolated bearing exchange to reconstruction of severe structural bone loss. Techniques for which the published experience remains anecdotal were not included.

## Search strategy

PubMed/MEDLINE and Scopus were searched for clinical studies, registry reports, systematic reviews, and biomechanical analyses relating to component retention in revision THA, published up to July 2026. Search terms combined "revision total hip arthroplasty" with "component retention", "dual mobility", "cemented liner", "cement-in-cement", "trunnion adapter", "head-neck adapter", "long cemented stem", and "cortical strut allograft". English-language articles reporting clinical outcomes, survivorship, or mechanical performance of the techniques considered were eligible. Single-patient case reports were excluded, and reference lists of included articles were hand-searched for additional sources. Older studies were retained where they represent the primary source for a technique. This process was structured but not systematic, and the article should be read as a narrative synthesis rather than a systematic review.

## Review of techniques and outcomes

### Retaining a MoM shell with dual mobility bearings

Explantation of a well-fixed MoM acetabular component is a technically demanding procedure associated with significant morbidity that frequently leads to substantial and undesirable loss of the existing bone stock, making subsequent reconstruction difficult [3, 4]. Retaining the shell and performing a single-component or bearing exchange revision offers a less invasive alternative that bypasses the morbidity of cup removal [5]. This bone-preserving strategy is particularly advantageous for young, active patients who typically possess good quality host bone [5]. For this approach to be indicated, the retained acetabular component must be well-fixed, appropriately positioned with adequate version and inclination (specifically, an abduction angle of less than 60°), and its internal geometry must be free from sharp edges or macroscopic surface damage [3, 6]. Moreover, this targeted retention method is highly applicable in managing specific mechanical failures, such as a femoral neck fracture of a resurfacing component; in these scenarios, the failed femoral side can be safely converted to a THA while leaving the secure acetabulum undisturbed [5].

The morbidity of full acetabular revision strongly supports this limited approach. In a direct comparative study, Colacchio et al. demonstrated a 20% major complication rate and a 16% re-revision rate for formal cup revisions, compared to a nearly five-fold decreased incidence of related re-operations when converting to a dual-mobility construct [6]. These constructs also offer significant perioperative clinical benefits compared to full component revision. Fishley et al. evaluated the exchange of metal heads for anatomical dual-mobility (ADM) or modular dual-mobility (MDM) polyethylene bearings while retaining well-fixed Birmingham MoM acetabular components [3]. Their study demonstrated that retaining the shell resulted in significantly shorter operative times (68.4 versus 101.5 min), reduced postoperative blood loss, and a shorter length of hospital stay (1.8 versus 2.4 days) compared to full acetabular revision. Importantly, patients were able to immediately and fully weight-bear postoperatively.

**Fig. 1:**
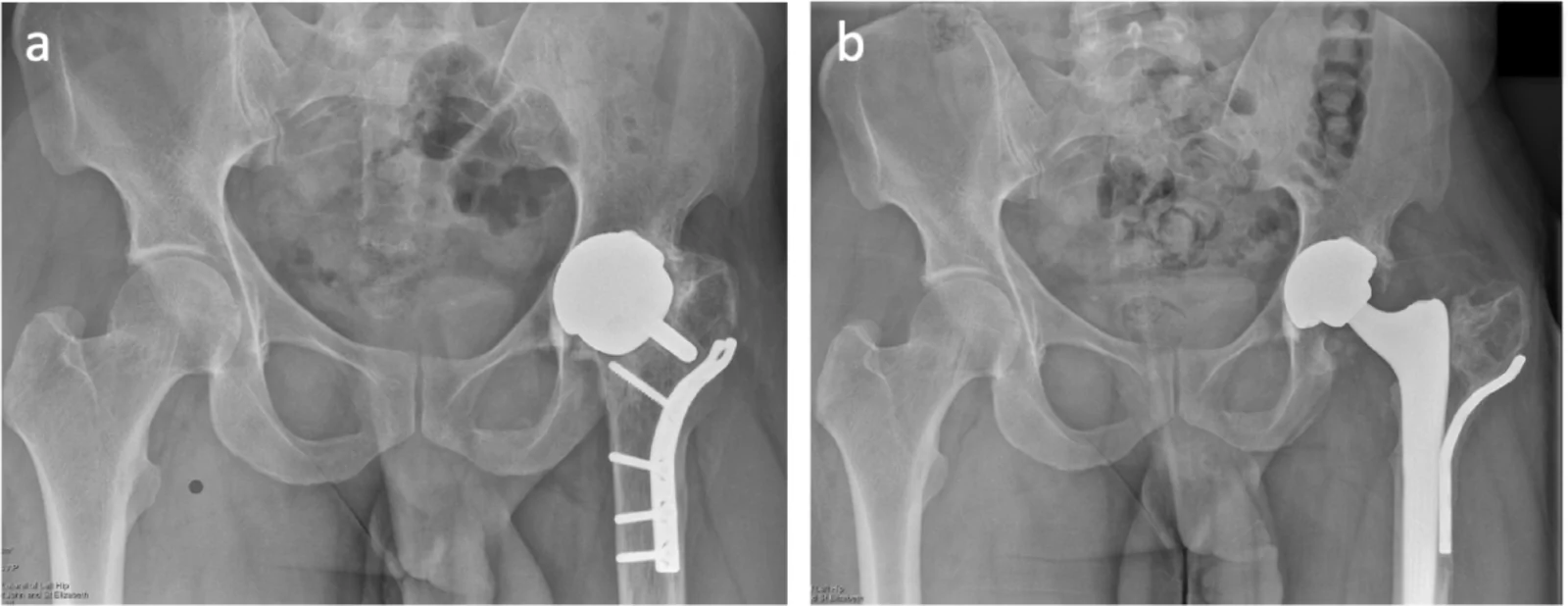
Anteroposterior pelvis radiographs of a failed hip resurfacing due to secondary avascular necrosis. **a** Preoperative radiograph showing the failed construct. **b** Postoperative radiograph following conversion to a total hip arthroplasty with retention of the well-fixed MoM acetabular shell

The risk of dislocation following revision THA for adverse reactions to metal debris is historically high [4], but dual-mobility constructs can effectively mitigate this risk. In their cohort, Fishley et al. reported a low 1.9% dislocation rate in the dual-mobility retention group [3]. This aligns with earlier findings by Figueras et al., who demonstrated that converting a MoM articulation to a dual-mobility head within a retained monoblock shell successfully restored hip stability, reduced ion levels, and preserved bone stock without instances of postoperative dislocation [5]. Additionally, mid-term follow-up in these dual-mobility retention cohorts has demonstrated no obvious radiographic signs of aseptic loosening, component migration, or accelerated polyethylene wear [6]. Other smaller series evaluating the salvage of monoblock MoM shells with dual-mobility bearings similarly report excellent mid-term survivorship and very low dislocation rates (ranging from 0 to 4%), supporting the feasibility of this interface-mixing strategy in selected patients, albeit in small retrospective series [7, 8] (Fig. 1).

### Cementing into revision shells

Expanding upon this pelvic preservation strategy, cementing a new bearing surface directly into a secure cup or existing cement envelope provides an alternative when standard modular component matching is precluded. Acetabular revision can be planned to avoid the bone loss and potential pelvic discontinuity associated with removing well-fixed components. When an existing acetabular shell or cement mantle is stable, retaining the shell and cementing a new liner provides a less invasive alternative. This strategy can involve cementing a liner into a retained metal shell, performing a cement-in-cement revision into an existing cement mantle, or cementing a dual-mobility liner into a newly placed porous revision shell [9–12].

Cementing a highly cross-linked polyethylene (XLPE) liner into a retained metal shell can be used when a compatible modular liner is unavailable or the original locking mechanism is damaged. To enhance fixation, the back surface of the liner is typically textured or engraved with a high-speed burr in a spider-web pattern to improve mechanical interdigitation with the bone cement [11]. The longest available follow-up is encouraging; Cho et al. reported a 93.3% implant survivorship free from re-revision at a mean follow-up of 14 years, noting zero instances of debonding at the liner-cement interface [11]. The primary cause of failure in their cohort was subsequent aseptic loosening of the pre-implanted outer metal shell rather than interface failure. Similarly, Xue et al. reported an 88.7% survivorship free from re-revision at a mean follow-up of 7.7 years [10]. Their multivariate analysis showed that a history of previous revisions was a significant risk factor for subsequent failure, and that patients revised for instability faced a higher risk of postoperative dislocation.

When revising a primary cemented acetabular component, a cement-in-cement technique can be used by retaining a secure cement mantle and cementing a new component into the existing void [9]. Brogan et al. reported the outcomes of 60 acetabular cement-in-cement revisions at a mean follow-up of 8.5 years, demonstrating a 100% five-year survival rate with aseptic loosening as the endpoint [9]. The surgical technique involves reaming out the old polyethylene liner using consecutive acetabular reamers while keeping the retained mantle undamaged, clean, and dry to ensure adequate interface shear strength before introducing the fresh cement [9].

**Fig. 2:**
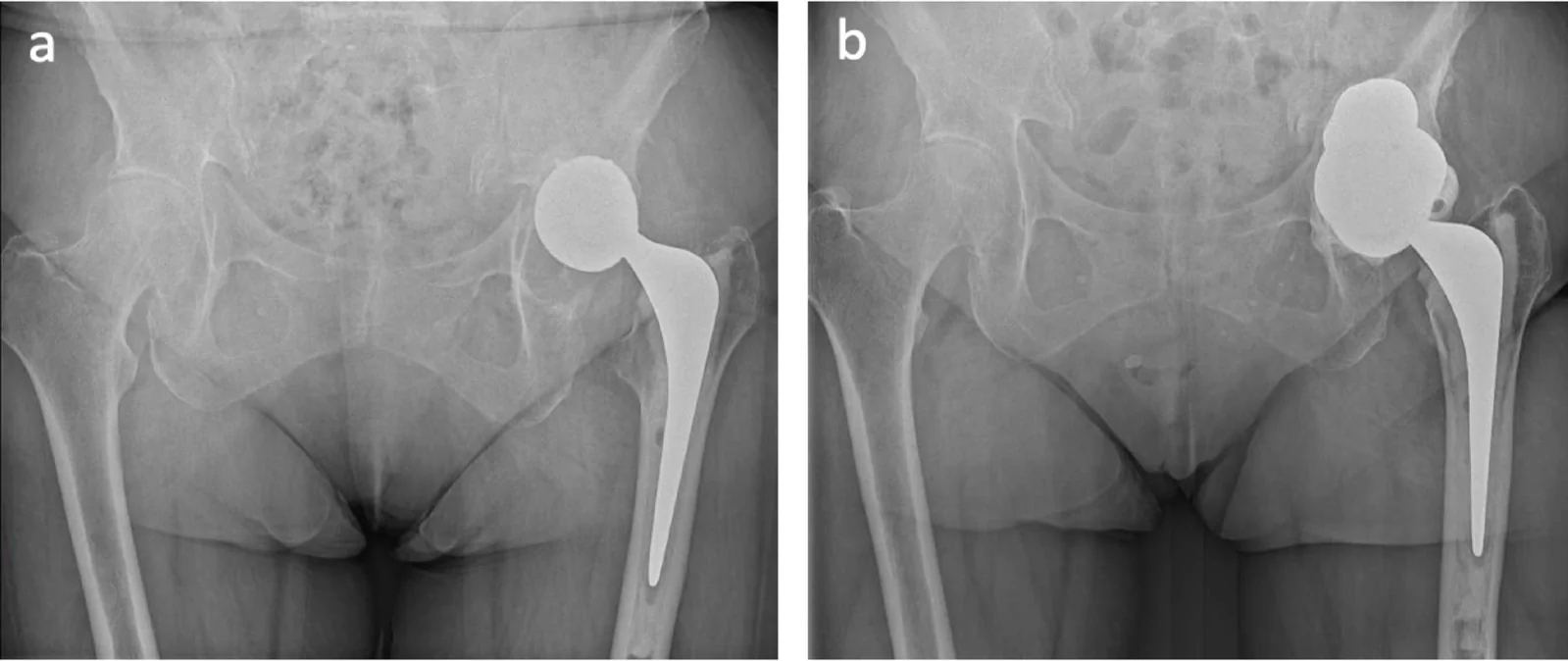
Anteroposterior pelvis radiographs demonstrating revision of a failed acetabular component with structural bone deficiency. **a** Preoperative view showing the failed construct. **b** Postoperative view following reconstruction using a porous revision shell with structural augments to optimize bone contact, into which a dual-mobility monoblock cup was cemented to independently control component orientation

Another variation of this approach involves cementing a dual-mobility liner into a porous revision shell to optimize construct geometry. Asaju et al. evaluated this technique in 47 revision total hip arthroplasties, highlighting its clinical utility in structurally challenging scenarios such as Paprosky type 2 and 3 defects [12]. By decoupling primary biological fixation from structural alignment, this method allows the surgeon to optimize bone contact and initial stability with the outer porous cup, while remaining free to independently control the abduction, anteversion, and overall orientation of the inner bearing (Fig. 2). At latest follow-up, their cohort showed no instances of postoperative dislocation or radiographic loosening, and the mean Oxford Hip Score improved from 24 preoperatively to 42 postoperatively [12].

### Stem retention utilizing trunnion adapters

While the preceding strategies address acetabular reconstruction, preserving a well-integrated femoral stem offers a parallel pathway to minimize surgical trauma. When an isolated bearing exchange is required, extracting a well-fixed femoral stem carries a significant operative burden and is associated with risks of perioperative morbidity, including cortical bone loss, prolonged operative times, and iatrogenic fracture. To bypass these complications and safely leave the stem in situ, changes can be made to the Morse taper junction; however, placing a new ceramic head directly onto a previously used trunnion risks stress concentration from preexisting mechanical or corrosive taper damage [13, 14]. This mismatch can lead to ceramic head fracture, mechanically assisted crevice corrosion, or accelerated fretting [13, 15].

Interposing a titanium adapter sleeve mitigates this risk, and the available retrospective data suggest reliable mid-term performance of this configuration. In a review of 516 revision THAs tracking fourth-generation ceramic heads with titanium sleeves on retained stems, Roberts et al. reported zero instances of ceramic head fracture, taper corrosion, or component disengagement at a mean follow-up of four years, achieving a 10-year survivorship free of re-revision of 85% [13]. Seah et al. reported on similar outcomes with no head-neck junction failures at a mean follow-up of 7.7 years in a series of 316 revisions, confirming that sleeved ceramic heads remain safe even when revising for adverse local tissue reactions [15]. Large-scale registry data evaluating 1,127 retained trunnions further suggested that although ceramic head fractures are exceptionally rare, an adapter sleeve facilitates safe head exchange without negatively impacting subsequent re-revision rates [14].

Beyond taper protection, specialized head-neck adapters, such as the BioBall Universal Adapter (Merete Medical, Germany), can also address independent biomechanical deficiencies [16]. Because instability is frequently driven by inadequate soft-tissue tension or component mismatch, these adapters allow surgeons to intraoperatively adjust femoral offset, anteversion, and leg length independently of the retained stem [16]. Garabadi et al. demonstrated the mechanical utility of this approach in an elderly cohort, where restoring offset and joint tension successfully resolved instability with only a single recurrent dislocation reported [16]. The adapter construct yielded a 98% survivorship alongside high patient-reported Forgotten Joint Scores, suggesting that it may be a reasonable option to optimise stability without stem extraction, although the reported series are small and retrospective [16]. However, adapter sizing can be restrictive, necessitating precise preoperative planning, and the technique remains contraindicated if there is gross malalignment of the existing stem [16]. Broader data support these findings. In a registry analysis of 354 revisions, Pardo et al. reported adapter survival of 87.9% at five years with no adapter or ceramic head breakage [17], and Hoberg et al. described 92.8% survival at 8.2 years in 95 consecutive cases [18]. However, a systematic review by Novoa et al. cautioned that the evidence remains Level IV and that re-revision rates are higher when the index indication is recurrent dislocation [19].

### Cement-in-cement femoral revision

**Fig. 3:**
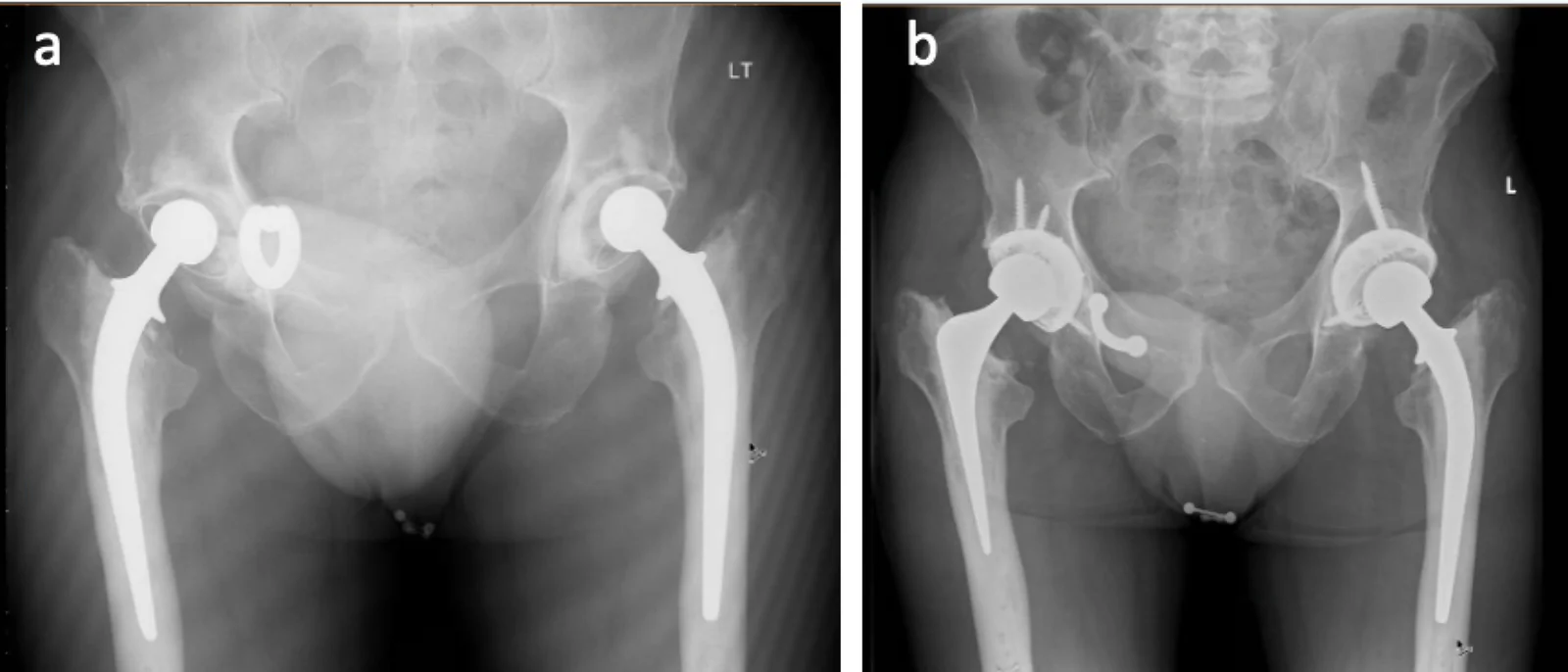
Anteroposterior radiographs of a right total hip arthroplasty undergoing a cement-in-cement femoral revision. **a** Preoperative view demonstrating a cemented femoral component within a structurally sound, well-integrated cement mantle. **b** Postoperative view following stem extraction and successful cement-in-cement reimplantation of a new femoral component within the retained cement matrix

When stem extraction becomes unavoidable but the underlying bone-cement interface remains sound, substituting the implant within the existing cement matrix offers an elegant, bone-preserving alternative (Fig. 3). The cement-in-cement technique involves extracting a femoral stem and cementing a new component into the existing intact cement void [20]. This strategy mitigates the substantial morbidity associated with complete cement extraction, including blood loss, extended operative times, and the high risk of iatrogenic cortical perforation or periprosthetic fracture [20, 21]. The primary prerequisite for this approach is a secure cement-to-bone bond; the technique is strictly contraindicated in cases demonstrating severe debonding across all Gruen zones [20, 22]. Preparation of the retained mantle routinely requires high-speed burrs or ultrasonic devices to selectively reshape the inner cement envelope, ensuring the precise seating of the new stem and optimal interdigitation of the fresh cement [20, 22].

The available clinical data indicate that cement-in-cement revision offers a faster and less costly alternative while maintaining satisfactory functional outcomes [20]. Malahias et al. demonstrated high implant survivorship and a low incidence of mechanical failure in a systematic review of aseptic revisions, provided the original cement mantle was structurally sound [22]. Deploying specifically designed short revision stems, such as the Exeter 44/00/125, further optimizes the procedure, yielding a 95.8% survivorship free from aseptic loosening at a mean follow-up of 8.6 years [23]. However, a rare but critical risk of prosthetic stem fracture remains. This risk is elevated when a short, undersized stem is cemented into a retained mantle in younger, heavier patients who lack adequate proximal bone support, creating a cantilever bending moment [24]. To mitigate this structural risk, utilizing components with higher mechanical thresholds, such as cobalt-chromium (CoCr) alloys, instead of traditional stainless steel has been proposed; general biomechanical and finite-element models of proximal femoral loading indicate that CoCr exhibits significantly higher yield strength and superior fatigue endurance limits when subjected to severe cyclic cantilever forces [25].

Beyond aseptic loosening and component malposition, the cement-in-cement technique can be applied to periprosthetic fractures around cemented stems in which the stem has become loose within its mantle but the cement–bone interface remains intact, corresponding to a Vancouver B2 fracture around a cemented implant [21]. The rationale is that the intact mantle and the surrounding cortex are used as a template: the fracture is anatomically reduced and provisionally held, most often with cerclage cables, and a longer revision stem is then cemented into the retained mantle so that it bypasses the fracture and acts as an internal splint. Because the original mantle is preserved, pre-fracture version, offset, and leg length can be reproduced directly, cement removal and its attendant risk of cortical perforation are avoided, and operative time and blood loss are reduced [21]. Care must be taken to seal the fracture line before pressurisation, as extrusion of fresh cement into the fracture site is a recognised cause of non-union and remains, together with concern over fixation stability, the principal reason the technique has not been more widely adopted [21]. Reported results are supportive: Briant-Evans et al. described radiographic union in all surviving cases of 23 Vancouver B fractures treated in this way [26], and Klasan et al. found no difference in five-year implant survival between in-cement revision and uncemented modular stem revision for B2 fractures around a cemented stem, with a mean saving of 45 min of operative time in the in-cement group [27].

### Long cemented stems

When proximal bone deficiency or structural damage is too extensive to permit a localized cement-in-cement revision, and yet profound osteopenia precludes standard cementless diaphyseal fixation, extending a new cemented construct distally offers an alternative. Historically, the use of cemented femoral stems in revision THA is associated with unacceptably high rates of mechanical failure and aseptic loosening, leading to the widespread adoption and current dominance of cementless diaphyseal-fitting components [28]. Despite this paradigm shift, long cemented stems remain a viable reconstructive option where immediate, reliable fixation is required and cementless purchase is unattainable [28, 29]. This applies particularly to elderly patients with profound osteopenia, where standard cementless revision stems lack sufficient diaphyseal purchase due to a lack of viable distal host bone [29, 30].

**Fig. 4:**
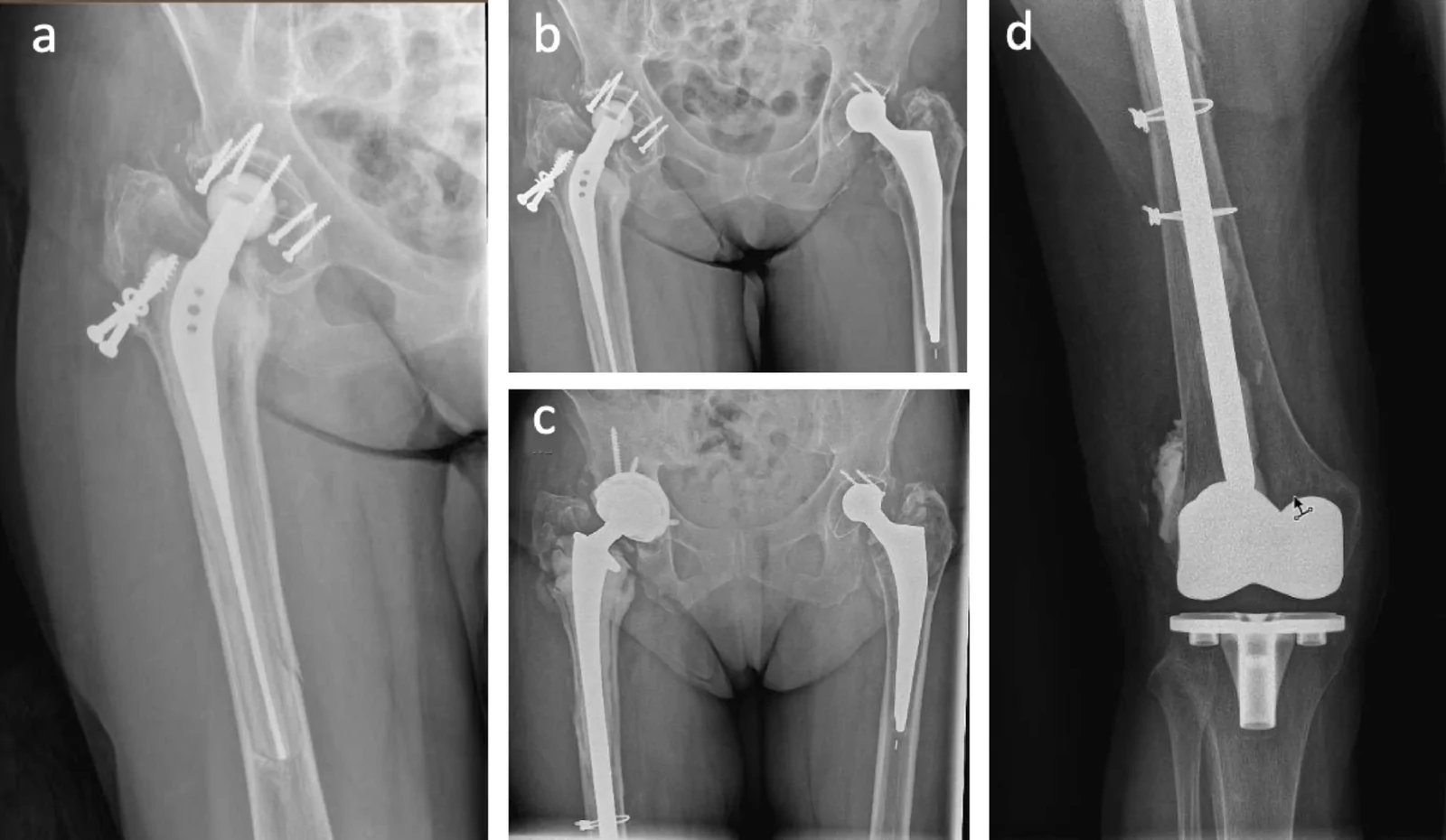
Radiographs demonstrating stabilization of a periprosthetic femoral fracture using an extended long cemented stem. Preoperative **a** femur and **b** pelvis radiographs showing a periprosthetic fracture in the setting of a structurally compromised, non-supportive diaphysis. Postoperative **c** pelvis and **d** distal femur radiographs following revision with a long cemented femoral prosthesis to bypass the diaphyseal defects and restore immediate stability

By bypassing major diaphyseal defects, this long-stem configuration achieves immediate stability [28, 29] (Fig. 4). Long-term clinical data support this strategy; in a series evaluating 37 consecutive fragile patients with a mean age of 76 years, te Stroet et al. demonstrated a 96.3% all-cause re-revision survival rate at a mean follow-up of nine years, with zero failures attributed to aseptic loosening [30].The biomechanics of composite beam stems, which bond tightly to the cement mantle to function as a single structural unit, provide distinct advantages in this geriatric population [31]. Deploying composite beam femoral components in these scenarios significantly reduces the rate of postoperative periprosthetic fractures compared to traditional taper-slip designs by distributing mechanical loads evenly across compromised cortical bone [31]. Furthermore, this structural rigidity permits immediate postoperative weight-bearing, which is a critical factor for reducing perioperative mortality and promoting functional recovery in the geriatric demographic [30].

Beyond mechanical stability, the substantial volume of cement required to seat a long-cemented stem serves as a local, high-dose antibiotic depot [28, 29]. This provides a distinct clinical advantage when revising hips with a history of periprosthetic joint infection or in immunocompromised patients requiring maximal infection prophylaxis [28, 29].

### Allograft and stem composites

**Fig. 5:**
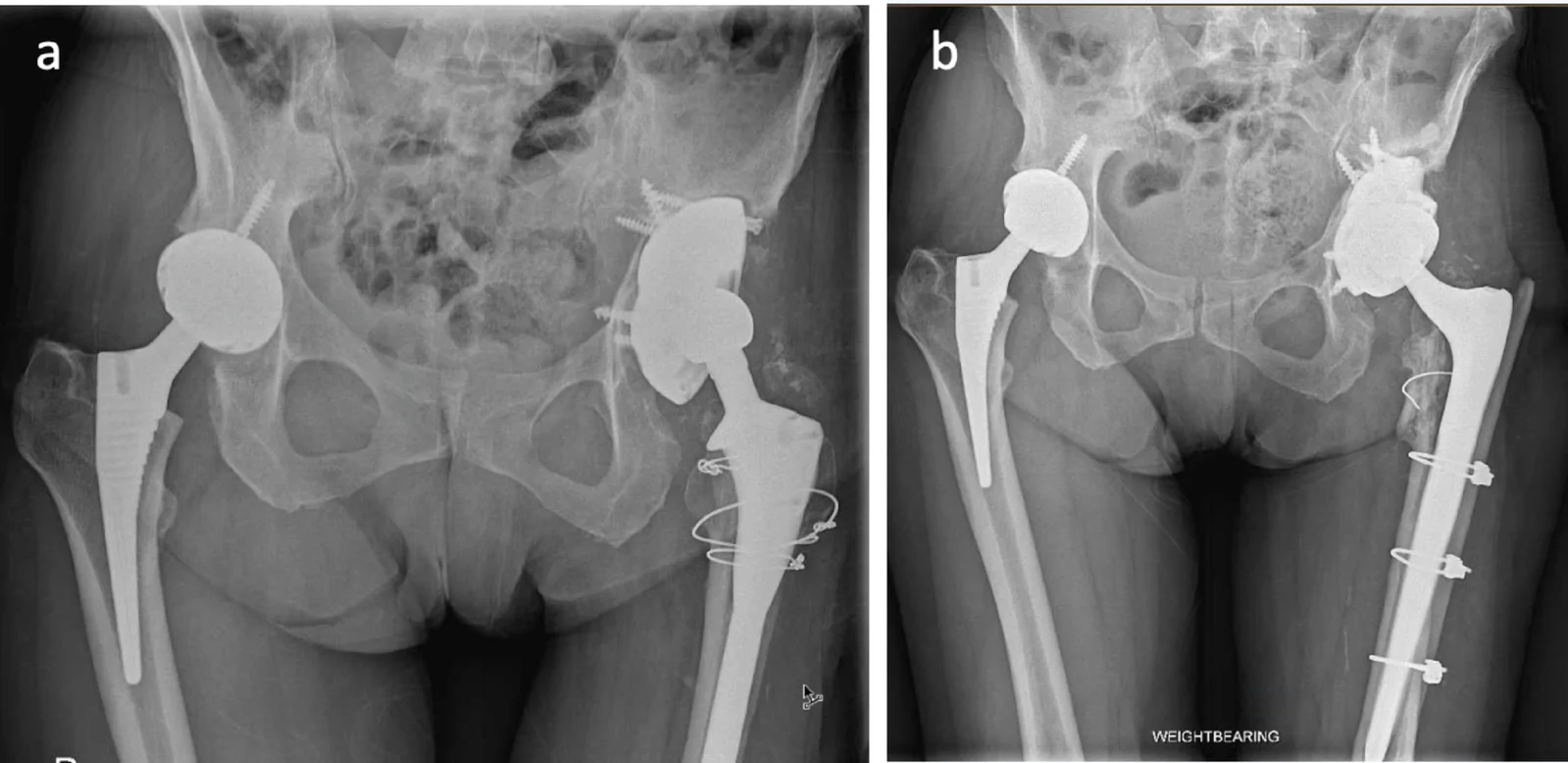
Anteroposterior radiographs of a left total hip arthroplasty undergoing reconstruction with a cortical strut allograft. **a** Preoperative view demonstrating a severely compromised proximal femur with critical thinning of the lateral cortex. **b** Postoperative view following revision utilizing a monoblock, cementless, diaphyseal-engaging femoral stem supplemented with a secured cortical strut allograft to bypass the structural defect, providing immediate mechanical rigidity and a biological scaffold for progressive bone restoration

When structural bone defects are too severe to be managed by extended hardware alone, biological reconstruction using onlay cortical strut allografts provides an alternative to purely mechanical bypassing [32] (Fig. 5). This approach reinforces a compromised cortical shell while preserving remaining host bone stock [32]. The strategy is typically divided into two configurations: combining struts with cemented long stems, or pairing them with cementless modular tapered fluted titanium (TFT) stems [32, 33].

Utilizing onlay cortical struts alongside a cemented femoral stem is primarily indicated for elderly patients presenting with severe osteopenia or expansive, thin-walled canals [33]. In this configuration, the internal cemented stem achieves immediate mechanical stability within the canal, while the contoured external struts are cabled tightly to the femur to function as biological plates [33]. Longitudinal data demonstrate that these struts progressively incorporate into the host skeleton through partial resorption and revascularization, thereby restoring proximal bone mass and decreasing construct strain over time [33, 34].

Alternatively, combining cortical struts with cementless components is used to manage severe proximal femoral deficiencies and associated periprosthetic fractures [32, 35, 36]. Evaluating this technique with proximally porous-coated titanium stems, Head et al. reported a 98% graft union rate with radiographic evidence of robust host-graft incorporation [36]. Their findings emphasize that the revision prosthesis itself must achieve rigid initial stability on viable host bone, relegating the external allograft to a role of structural augmentation rather than primary prosthetic support [36]. Modern applications utilize splined, modular TFT stems to achieve scratch-fit stability in the distal diaphysis [35]. Simultaneously, the external cortical struts are cabled across the proximal femur to neutralize rotational forces and span modular junctions, protecting the hardware from fatigue fractures at high-stress interface points [32]. By providing a biological scaffold that fuses with the host cortex, this technique allows for progressive proximal stress distribution, reducing distal stress shielding and preserving bone stock [32, 35].

## Critical appraisal of the evidence

The evidence supporting these strategies is uneven. Most of the cited work consists of retrospective, single-centre cohorts and systematic reviews of such cohorts, corresponding to Level III and IV evidence, and several series are small. Follow-up varies considerably between techniques, from a mean of four years for ceramic heads with titanium sleeves on retained stems to fourteen years for cemented cross-linked polyethylene liners, so survivorship figures are not directly comparable across sections. Registry data provide broader denominators for trunnion re-use and cement-in-cement femoral revision, but lack the granularity to confirm the indication for, or the technical execution of, each procedure. Indications are also highly selective by design, since these techniques are only applicable when the retained component is well fixed and appropriately positioned; outcomes therefore cannot be extrapolated to unselected revision THA populations. Finally, no randomised or matched comparative study has yet tested targeted retention against complete explantation for most of the techniques described, with the comparative cohorts of Fishley et al. [3] and Colacchio et al. [6] being notable exceptions on the acetabular side. The conclusions of this review should be interpreted with these constraints in mind, and longer-term survivorship and PROM data remain necessary.

## Summary

**Fig. 6:**
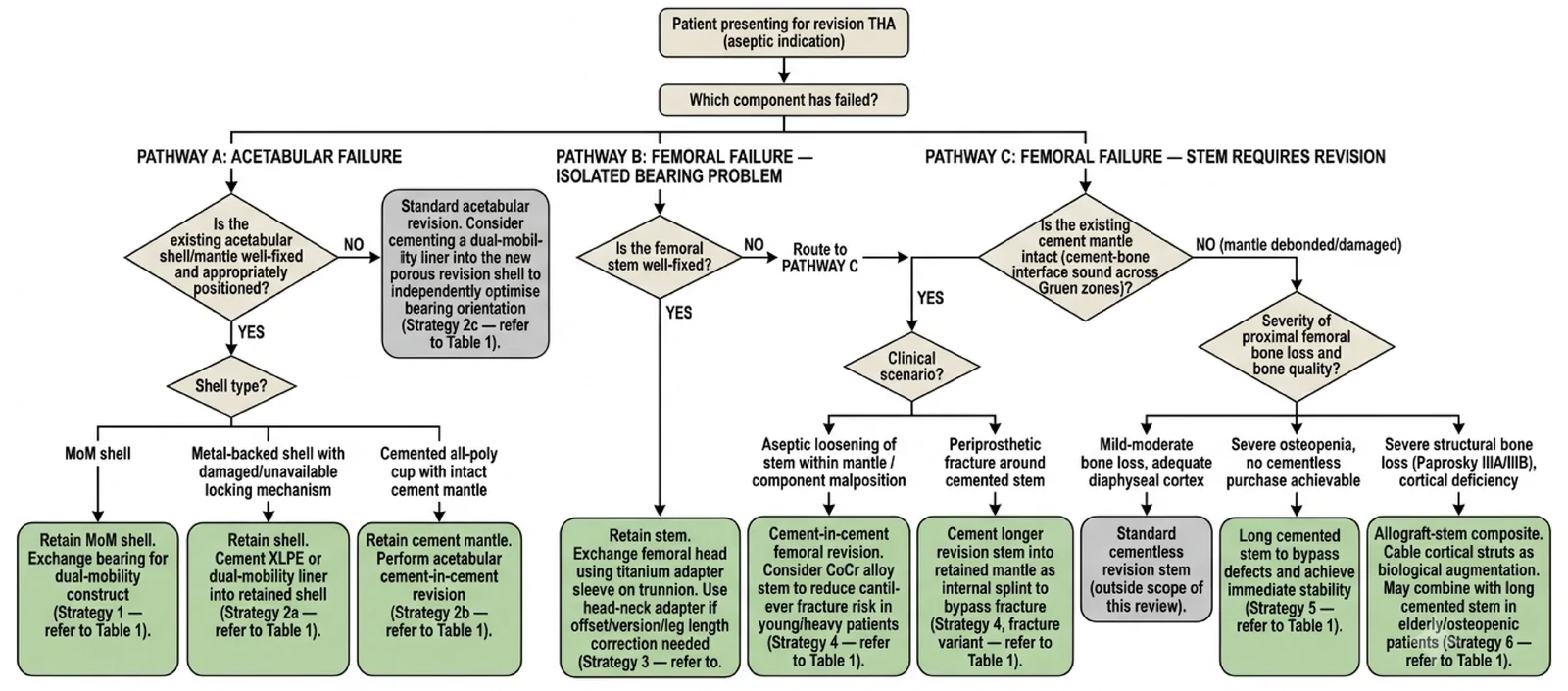
Treatment algorithm for targeted component retention in revision total hip arthroplasty. This algorithm applies to aseptic indications only. Green boxes indicate component-retention strategies reviewed in this manuscript (Strategies 1–6, cross-referenced to Table 1); grey boxes indicate standard revision pathways included for context. *MoM* Metal-on-metal, *XLPE* Highly cross-linked polyethylene, *CoCr* Cobalt-chromium

A comparative synthesis of the six strategies, including indications, contraindications, reported survivorship, and level of evidence, is presented in Table 1. A decision-making algorithm based on component fixation status, cement mantle integrity, and the extent of femoral bone loss is shown in Fig. 6. The six strategies described in this review reflect a shared operative philosophy: that the preservation of well-fixed, appropriately positioned components reduces surgical morbidity without compromising clinical outcomes. Retaining a stable MoM acetabular shell with a dual-mobility bearing avoids the substantial bone loss of formal cup explantation while delivering perioperative benefits and low dislocation rates. Additionally, cementing XLPE or dual-mobility liners into revision or retained shells permits independent optimisation of bearing orientation, yielding acceptable survivorship across a range of acetabular defect classifications. On the femoral side, trunnion adapter sleeves facilitate safe head exchange on retained femoral stems, with registry and institutional cohort data suggesting durability of this construct at up to ten years, although prospective evidence is lacking. The cement-in-cement technique similarly provides a fast and reliable revision pathway when the original cement mantle remains structurally intact, with demonstrated long-term survivorship and utility even in the context of periprosthetic fracture and infection. Finally, long cemented stems and allograft-stem composites address the more complex reconstructive challenge of severe structural bone loss, offering immediate stability in osteopenic femurs where cementless diaphyseal purchase is unachievable, and providing a biological scaffold for progressive proximal stress restoration. Functional outcome data are reported less consistently than survivorship across these techniques, and the instruments used differ between series, which precludes pooling. Where PROMs are available, the results are favourable: cementing a dual-mobility liner into a porous revision shell improved the mean Oxford Hip Score from 24 to 42 [12], head-neck adapters used to restore offset and soft-tissue tension yielded high Forgotten Joint Scores alongside 98% survivorship [16], and ceramic heads with titanium sleeves on retained stems achieved satisfactory functional scores at mid-term follow-up [13]. Several of the remaining series report survivorship and radiographic outcomes without formal PROMs. Standardised, prospectively collected PROM reporting therefore remains an important gap and should be prioritised in future studies of component retention.

**Table 1:** Comparative synthesis of six targeted component-retention strategies in revision total hip arthroplasty

Strategy	Key Indications	Key Contraindications	Survivorship	Level of Evidence
1. MoM shell retention with dual-mobility bearing	Well-fixed, well-positioned MoM shell (abduction < 60°); failed resurfacing with stable cup [3–6]	Shell loosening, malposition, macroscopic surface damage, active infection [3, 6]	96–100% at 3–6 yr; dislocation 0–4% [3, 5–8]	III–IV
2a. Cementing liner into retained metal shell	Stable metal-backed shell with unavailable/damaged modular liner mechanism [10, 11]	Shell loosening or malposition [10, 11]	88.7–93.3% at 7.7–14 yr [10, 11]	III–IV
2b. Acetabular cement-in-cement	Stable cemented acetabular mantle requiring liner exchange [9]	Mantle debonding or structural compromise [9]	100% at 5 yr (aseptic loosening endpoint) [9]	IV
2c. Dual-mobility liner cemented into porous revision shell	Complex acetabular defects (Paprosky 2–3); need to decouple fixation from bearing orientation [12]	Inadequate shell-bone contact for biological fixation [12]	100% implant retention at mid-term follow-up [12]	IV
3. Trunnion adapter / head-neck adapter on retained stem	Isolated bearing exchange with well-fixed stem; offset/version correction needed [13–16]	Gross stem malalignment; severely damaged trunnion [13, 16]	85–98% at 7–10 yr [13–16]	III–IV
4. Cement-in-cement femoral revision	Intact cement mantle with stem failure; periprosthetic fracture around cemented stem [20–22]	Severe mantle debonding across all Gruen zones; active infection (relative) [20, 22]	95.8% at 8.6 yr (aseptic loosening endpoint) [23]	III–IV
5. Long cemented stem	Severe osteopenia precluding cementless fixation; elderly patients; need for antibiotic depot [28–30]	Adequate diaphyseal bone supporting cementless purchase [28]	96.3% at 9 yr (all-cause re-revision) [30]	IV
6. Allograft-stem composite	Severe structural bone loss (Paprosky IIIA/IIIB); cortical deficiency requiring biological reconstruction [32, 35]	Insufficient host bone for primary prosthetic fixation; active infection [36]	98% graft union rate [36]	IV

*MoM* Metal-on-metal, *yr* Years

Collectively, these strategies challenge the notion that revision THA mandates wholesale component removal. Their appropriate application requires precise preoperative planning, thorough intraoperative assessment of component stability, and an understanding of the biomechanical demands specific to each clinical scenario. Furthermore, establishing more robust evidence regarding long-term clinical survivorship, complication profiles, and PROMs remains imperative to fully validate these strategies. As revision burden continues to grow with an ageing arthroplasty population [37], familiarity with and the continuous scientific tracking of targeted retention techniques will be increasingly central to achieving the dual goals of reduced operative morbidity and durable long-term reconstruction.

## Data Availability

No datasets were generated or analysed during the current study.

## References

[1] Dubin J, Westrich G (2022) Differences in patient-reported outcome measures between primary and revision total hip arthroplasty: realistic patient expectations for patients with low baseline activity. Orthopedics 45:251–255 10.3928/01477447-20220225-04 35245136

[2] Mohammad O, Shaarani S, Mohammad A et al (2024) Patients’ expectations surrounding revision total hip arthroplasty: a literature review. Arthroplast. 10.1186/S42836-024-00250-6 PMC11145824 38825694

[3] Fishley W, Nandra R, Carluke I et al (2024) Revision of metal-on-metal hip replacements with dual-mobility bearings and acetabular component retention. Bone Jt Open 5:514–523 10.1302/2633-1462.56.BJO-2023-0165.R1 PMC11194627 38910515

[4] Chang JS, Haddad FS (2020) Revision total hip arthroplasty for metal-on-metal failure. J Clin Orthop Trauma 11:9–15 10.1016/j.jcot.2019.09.021 PMC6985013 32001977

[5] Figueras G, Planell RV, Fernàndez RS et al (2016) Revision of metal-on-metal hip arthroplasty with well fixed and positioned acetabular component using a dual-mobility head and review of literature. Open Orthop J 10:512–521 10.2174/1874325001610010512 PMC5093867 27857822

[6] Colacchio ND, Wooten CJ, Martin JR et al (2020) Dual mobility for monoblock metal-on-metal revision—is it safe? J Arthroplasty 35:508–512 10.1016/j.arth.2019.09.028 31662280

[7] Plummer DR, Botero HG, Berend KR et al (2016) Salvage of monoblock metal-on-metal acetabular components using a dual-mobility bearing. J Arthroplasty 31:846–849 10.1016/j.arth.2015.08.016 26404847

[8] Klemt C, Smith EJ, Oganesyan R et al (2020) Outcome of dual mobility constructs for adverse local tissue reaction associated abductor deficiency in revision total hip arthroplasty. J Arthroplasty 35:3686–3691 10.1016/j.arth.2020.06.043 32654942

[9] Brogan K, Charity J, Sheeraz A et al (2012) Revision total hip replacement using the cement-in-cement technique for the acetabular component: technique and results for 60 hips. J Bone Joint Surg Br 94-B:1482–1486 10.1302/0301-620X.94B11.29415 23109626

[10] Xue Z, Ding Z, Mu W et al (2025) Cementing liners into well-fixed acetabular components in revision total hip arthroplasty: a mean 7.7-year follow-up. J Arthroplasty 40:S83–S90 10.1016/j.arth.2025.07.047 40714211

[11] Cho K, Park CW, Jeong SJ et al (2023) Long-term outcomes of cementing highly cross-linked polyethylene liners into well-fixed acetabular shells in revision total hip arthroplasty. J Arthroplasty 38:1335–1341 10.1016/j.arth.2023.01.028 36709880

[12] Asaju AO, Shaarani S, Shaeir M et al (2026) Outcome of dual-mobility articulation in revision hip arthroplasty of porous revision acetabular component. Hip Int. 10.1177/11207000251372023 41169170

[13] Roberts HJ, Hannon CP, Dilger OB et al (2024) New ceramic heads with titanium sleeves on retained femoral components: results of over 500 revision total hip arthroplasties. J Arthroplasty 39:S183–S187 10.1016/j.arth.2024.01.045 38336305

[14] Affatato S, Cosentino M, Castagnini F et al (2019) Registry study on failure incidence in 1,127 revised hip implants with stem trunnion re-use after 10 years of follow-up: limited influence of an adapter sleeve. Acta Orthop 90:417–420 10.1080/17453674.2019.1618649 PMC6746291 31210073

[15] Seah MKT, Howard LC, Horwood NJ et al (2025) Is the use of new ceramic heads with titanium sleeves on retained femoral stems in revision total hip arthroplasty associated with femoral head or neck junction failure at mean eight-year follow-up? J Arthroplasty. 10.1016/J.ARTH.2025.11.058 41318036

[16] Garabadi M, Akhtar M, Blow J et al (2023) Clinical outcome of Bioball universal adapter in revision hip arthroplasty. J Orthop 38:68–72 10.1016/j.jor.2023.02.015 PMC10063389 37008449

[17] Pardo F, Castagnini F, Bordini B et al (2023) A modular head-neck adapter system and ceramic heads in revision hip arthroplasty: a registry study on 354 implants. J Arthroplasty 38:1580–1585 10.1016/j.arth.2023.01.055 36764407

[18] Hoberg M, Konrads C, Huber S et al (2015) Outcome of a modular head-neck adapter system in revision hip arthroplasty. Arch Orthop Trauma Surg 135:1469–1473 10.1007/s00402-015-2281-z 26187599

[19] Novoa CD, Citak M, Zahar A et al (2018) The Merete BioBall system in hip revision surgery: a systematic review. Orthop Traumatol Surg Res 104:1223–1228 10.1016/j.otsr.2018.06.016 30391216

[20] Kumar A, Porter M, Shah N et al (2019) Outcomes of cement in cement revision, in revision total hip arthroplasty the creative commons attribution-NonCommercial 4.0 International License (CC BY-NC 4.0). Maced J Med Sci 7:4059–4065 10.3889/oamjms.2019.710 PMC7061388 32165952

[21] Wood MJ, Al-Jabri T, Mirdad RS et al (2026) Cement-in-cement revision of the cemented femoral stem as a treatment for periprosthetic fracture around a total hip arthroplasty: an up-to-date review. Injury 57:112932 10.1016/j.injury.2025.112932 41442906

[22] Malahias M-A, Mancino F, Agarwal A et al (2021) Cement-in-cement technique of the femoral component in aseptic total hip arthroplasty revision: a systematic review of the contemporary literature. J Orthop 26:14–22 10.1016/j.jor.2021.06.002 PMC8261467 34276146

[23] Woodbridge AB, Hubble MJ, Whitehouse SL et al (2019) The Exeter short revision stem for cement-in-cement femoral revision: a five to twelve year review. J Arthroplasty 34:S297–S301 10.1016/j.arth.2019.03.035 31000404

[24] Bolland BJRF, Wilson MJ, Howell JR et al (2017) An analysis of reported cases of fracture of the universal Exeter femoral stem prosthesis. J Arthroplasty 32:1318–1322 10.1016/j.arth.2016.09.032 27843041

[25] Cakir F, Özkal FM, Sensoz E (2022) Performance assessment of biocompatible metals used in the treatment of femoral neck fractures. ACS Appl Bio Mater 5:3013–3022 10.1021/acsabm.2c00321 PMC9214763 35674244

[26] Briant-Evans TW, Veeramootoo D, Tsiridis E et al (2009) Cement-in-cement stem revision for Vancouver type B periprosthetic femoral fractures after total hip arthroplasty: a 3-year follow-up of 23 cases. Acta Orthop 80:548–552 10.3109/17453670903316827 PMC2823329 19916687

[27] Klasan A, Millar J, Quayle J et al (2022) Comparable outcomes of in-cement revision and uncemented modular stem revision for Vancouver B2 periprosthetic femoral fracture at 5 years. Arch Orthop Trauma Surg 142:1039–1044 10.1007/s00402-021-03776-5 33575925

[28] Roussot MA, Vles GF, Haddad FS (2018) The role of cemented stems in revision total hip arthroplasty. Semin Arthroplasty 29:177–182

[29] Hoveidaei AH, Pirahesh K, Sezgin EA et al (2025) Can cemented femoral stems be used during revision total hip arthroplasty? J Arthroplasty 40:S176–S179 10.1016/j.arth.2024.10.069 39437861

[30] Te Stroet MAJ, Bronsema E, Rijnen WHC et al (2014) The use of a long stem cemented femoral component in revision total hip replacement: a follow-up study of five to 16 years. Bone Joint J 96-B:1207–1213 10.1302/0301-620X.96B9.33304 25183592

[31] Mili-Schmidt V, Magnéli M, Logishetty K et al (2026) Reduced long-term periprosthetic fracture rates with composite beam versus polished tapered stems in cemented hip arthroplasty a ten-year observational study. Bone Jt Open 7:316–325 10.1302/2633-1462.73.BJO-2025-0350 PMC12964061 41788013

[32] Lim CT, Amanatullah DF, Huddleston JI et al (2017) Cortical strut allograft support of modular femoral junctions during revision total hip arthroplasty. J Arthroplasty 32:1586–1592 10.1016/j.arth.2016.12.011 28130016

[33] Emerson RH, Malinin TI, Cuellar AD et al (1992) Cortical strut allografts in the reconstruction of the femur in revision total hip arthroplasty. A basic science and clinical study-PubMed. Clin Orthop Relat Res 285:35–44 1446451

[34] Haddad FS, Garbuz DS, Masri BA et al (2000) Structural proximal femoral allografts for failed total hip replacements: a minimum review of five years. J Bone Joint Surg Br 82:830–836 10.1302/0301-620x.82b6.10485 10990306

[35] Mayle RE, Paprosky WG (2012) Massive bone loss: allograft-prosthetic composites and beyond. J Bone Joint Surg Br 94:61–64 10.1302/0301-620X.94B11.30791 23118384

[36] Head WC, Wagner RA, Emerson RH et al (1994) Revision total hip arthroplasty in the deficient femur with a proximal load-bearing prosthesis. Clin Orthop Relat Res 298:119–126 8118966

[37] Shichman I, Askew N, Habibi A et al (2023) Projections and epidemiology of revision hip and knee arthroplasty in the United States to 2040–2060. Arthroplast Today 21:101152 10.1016/j.artd.2023.101152 PMC10244911 37293373

